# Meat consumption among different social groups and specific options for reducing it: a literature review of empirical research

**DOI:** 10.3389/fsoc.2025.1547663

**Published:** 2025-05-23

**Authors:** Thea Xenia Wiesli

**Affiliations:** Faculty of Social and Political Sciences, Department of Sociology, University of Innsbruck, Innsbruck, Austria

**Keywords:** consumption behavior, sustainable nutrition, social groups, social status, social class, plant based nutrition

## Abstract

The overconsumption of meat, and the connected overproduction of meat, contribute significantly to climate change, deforestation, biodiversity loss, and public health risks. There is a need to reduce global meat consumption. On average, high- and middle-income countries have the highest levels of meat consumption. However, within individual societies, social groups and classes differ in their food habits and *ability to engage in sustainable nutritional habits*. This literature review provides information on how socio-economic characteristics, social status, norms, and structural context shape meat consumption, and what interventions can effectively reduce specific social groups' meat consumption. Empirical studies published between 2019 and 2024 were researched and screened, adopting the PRISMA approach. The findings highlight critical variations in meat consumption by gender, age, social status, social norms, and context effects. Effective interventions include tailored approaches such as price incentives, normative messaging, and increasing the accessibility of plant-based options. The discussion underscores the importance of policymakers and stakeholders applying targeted and status-sensitive strategies to support sustainable dietary shifts and to address social inequalities.

## 1 Introduction

The overconsumption of meat, alongside massive meat production, leads to considerable environmental (Domingo et al., 2021), climate (Xu et al., 2021) and social challenges (Grethe, 2017; Muller, 2018; OECD, 2023). Meat production is a crucial cause of climate warming. Compared to plant-based food, meat production is responsible for twice the amount of greenhouse gas emissions, and thus exacerbates climate warming (Xu et al., 2021). Industrial livestock farming and the overconsumption of meat pose several physical health risks to humans. Air and water pollution, a frequent consequence of industrial livestock farming, present risks to animal and human health (Domingo et al., 2021; Gilbert et al., 2021). Most antibiotics worldwide are used for livestock farming. Thus, industrial livestock farming increases the risk of increased antimicrobial resistance among animals and humans (Espinosa et al., 2020). The physical and genetic proximity of animals bred and held en masse also presents conditions favorable to the emergence of diseases such as zoonoses (Espinosa et al., 2020; Yang et al., 2016). Meat production is also one of the leading causes of global deforestation and biodiversity loss (Henry et al., 2019; Machovina et al., 2015).

Considering food insecurity in low-income nations and the growing global population, it is inefficient to use two-thirds of global agricultural land for grazing and feed; large parts of this can be used to cultivate plant-based food instead and thus directly meet human nutritional needs^1^ (FAO, 2020). The current situation challenges food justice on a global level, particularly because global meat consumption is predicted to continue rising until 2050 due to increasing economic and population growth (FAO, 2018; OECD/FAO, 2021). In middle-income countries with significant economic growth, consumption increases substantially (OECD, 2023). In high-income countries, meat consumption stagnates or declines (FAO, 2018). Nevertheless, the annual meat consumption of high-income countries is approximately 80–100 kg per person, while middle-income countries consume around 30–50 kg per person (OECD, 2023). A sustainable meat consumption level is approximately 15–20 kg per year per person, significantly lower than current consumption levels in high-income countries (EATR, 2019). Shifting dietary patterns, and particularly reducing meat consumption, is a critical component of the socioecological transformation, with the potential to decrease resource use, lower greenhouse gas emissions, and address social inequalities simultaneously. Furthermore, it can potentially reduce ever-increasing nutrition-related health costs.

Levels of meat consumption differ not only on the global level but also on the societal level, and thus between social groups. Dietary behavior is part of lifestyles, with personal routines shaped by a complex interplay of contextual conditions and social, cultural, and economic factors (Paddock, 2017; Prahl and Setzwein, 1999; Schleicher and Toeller, 2024). As social groups within societies differ in their preferences and capabilities regarding diets, there is a need to know how meat consumption differs among social groups and what the specific options are for interventions to achieve sustainable levels of meat consumption. Although the reasons for the particular consumption patterns of social groups do not always lead to motivations or possibilities to reduce meat consumption, knowledge about these reasons allows us to address social groups with targeted measures.

Literature reviews from different disciplines show individuals' consumption of meat (Benningstad and Kunst, 2020; da Veiga et al., 2023; Modlinska and Pisula, 2018) and plant-based food (e.g., Akinmeye et al., 2024; Nguyen et al., 2022; Onwezen, 2022; Rickerby and Green, 2024; Szenderak et al., 2022), attitudes regarding meat (e.g., Moosburger et al., 2023; Pohlmann, 2022; Sanchez-Sabate et al., 2019; Valli et al., 2019; Wang, 2022), the potential for reducing meat consumption (Bianchi et al., 2018a,b; Blezins Moreira et al., 2022; Kwasny et al., 2022; Shimokawa, 2015; Viroli et al., 2023) and options to enhance sustainable nutrition (e.g., Blackford, 2021; Elliott et al., 2024; Hoek et al., 2021; Pandey et al., 2023). These literature reviews cover societies in general. Exceptionally, Stoll-Kleemann and Schmidt (2017) published a literature review on socio-economic groups' behavior, barriers, opportunities and steps that need to be taken to encourage less meat consumption, from a psychological perspective.

However, there is currently no literature review that covers recent empirical findings on meat consumption by specific social groups or classes, and specific intervention options for reducing meat consumption. This information is needed in order to develop further research on knowledge about the meat consumption of different social groups, their different statuses, and targeted support actions. Additionally, such an overview is also important for policymakers seeking to apply measures that can be empirically shown to significantly support social groups in reducing meat consumption in a tailored way.

This literature review summarizes recent empirical work in middle- and high-income countries published between 2019 and 2024 to fill this knowledge gap and extend previous literature reviews. The findings present the meat consumption habits of social groups that differ by age, gender, family status, social status, and norms, as well as specific intervention options for reducing meat consumption.

The following section briefly introduces the most important social science theories regarding the social distinction of meat consumption.

### 1.1 The social distinction of meat consumption

Several sociologists have described how social groups distinguish themselves from one another by goods, behavior, and taste. For example, more than a century ago, Veblen described in *The Theory of the Leisure Class* (Veblen, 2017) how elite social groups consume specific, often pricy, goods without practical function to distinguish themselves from other groups.

Bourdieu famously stated in *Distinction: A Social Critique of the Judgment of Taste* that people acquire a class-specific habitus through socialization (1984). Accordingly, social classes differ in their economic, cultural and social capital. Economic capital involves financial and material wealth that provides access to goods and services. Cultural capital encompasses education, skills, and cultural knowledge that influences behavior and social status. Social capital includes social networks and connections that provide support and access to resources. Regarding dietary behavior, Bourdieu (1984) demonstrated in his studies from 1963 to 1979 in France that social classes differ in their preferences for food and the way and situations in which they prepare food and eat, based on their life circumstances. According to Bourdieu, the upper classes distinguish themselves from the lower classes by eating lighter, more exquisite, more original, or exotic food. Furthermore, food is aesthetically arranged when served. Lower classes tend to eat more filling food, often served directly from a pot on the table. Their taste is shaped by necessity and adaptation to the given life conditions (Bourdieu, 1984).

Meat has a particular social significance, symbolizing affiliation with or demarcation from other social groups, and is deeply embedded in many food cultures (Fiddes, 1994; Prahl and Setzwein, 1999). A widespread explanation for this fact is the historical difficulty of obtaining meat, which made it expensive and a symbol of high status or even power (Spencer, 1996), and the fact that people imitate the food choices of their social companions (Fiddes, 1994; Higgs and Ruddock, 2020) or higher classes (e.g., Bourdieu, 1984). Other explanations focus on the culture and history of agriculture and countries' culinary heritage (Chiles and Fitzgerald, 2018). In many countries ranking near the top with regard to the level of meat consumption, such as Argentina, the United States, Australia and Saudi Arabia, pastoral farming has historically been the predominant form of farming. Pastoral farming in this context includes extensive animal husbandry on grazing land, often for cattle, goats, sheep, or other livestock, due to the low agricultural potential of the land, such as steppes, savannas, or dry highland areas. This led to the keeping of cows for beef and is probably one of the reasons why the barbecue is an important cultural and social custom in Australia, the United States and Argentina.

Elias (2000) pointed out in *The Civilizing Process* (1969) that the consumption and preparation of meat gradually became more ritualized and was surrounded by rules designed to minimize the visibility of violence or blood, as well as to control emotional reactions associated with eating meat. Throughout this process, the high status of meat as an element of a meal remained. Douglas and Isherwood (1979) famously pronounced in her research in the 1970s in England that the average consumer defines a “proper meal” by counting the components. Meat is the center, and vegetables and food that is high in carbohydrates are the side dishes. This perception of a proper meal is still widespread in Western cultures (Astleithner, 2007; Purhonen and Gronow, 2014). Moreover, meat is connected to rituals and traditions, is served on festive occasions, and signifies hospitality (Astleithner, 2007; Fiddes, 1989; Purhonen and Gronow, 2014).

More recent concepts, such as “eco habitus” and “(cultural) omnivores,” indicate that sustainable nutrition is also a way of gaining distinction, and self-identification, and does not only reflect people's awareness of environmental issues. For example, Kristóf and Megyesi (2024) demonstrate, with their qualitative results in Hungary, how more affluent and educated groups perceive sustainable food as a personal expression, a mark of status, and as a part of modern aesthetics, and choose it as a lifestyle. In contrast, social groups with limited resources tend to adopt sustainable practices primarily for their practicality, affordability, and consistency with traditional lifestyles (Kristóf and Megyesi, 2024). Other studies have suggested that ethical consumption (Kennedy et al., 2018), veganism, and vegetarianism (Kennedy et al., 2018; Rosenfeld et al., 2020) act as an expression of high status (Rosenfeld et al., 2020). One reason for this might be that higher income correlates with environmentally conscious dietary choices (Stoll-Kleemann and Schmidt, 2017). This interpretation of status regarding diets indicates that there are indirect reasons for consuming meat, which lead to different effects depending on the social group.

## 2 Method

The method applied in preparing this article was a literature review. The review mainly applied the PRISMA approach (Preferred Reporting Items for Systematic Reviews and Meta-Analyses), with some exceptions. The PRISMA approach is a widely recognized methodology for conducting and reporting systematic reviews and meta-analyses. It provides a framework for ensuring transparency, reproducibility, and comprehensiveness in reviewing research evidence.

Unlike the standard PRISMA approach, independent reviewers were not involved in the screening process for this review, and a formal bias assessment of the included studies was not conducted. However, the inclusion criteria were predefined, and the study selection process was reviewed and cross-checked against these criteria at several stages. A protocol and the corresponding decision-making process were carefully reconsidered in cases of uncertainty to ensure consistency and objectivity. Inclusion criteria, such as the requirement for peer-reviewed publications and adherence to empirical research standards, were applied to minimize the likelihood of significant methodological biases.

The author searched for and screened studies between March and August 2024. The predefined inclusion criteria were carefully followed, and the study selection process was reviewed and cross-checked against these criteria at multiple stages. A detailed description of the process is provided below (see Section 2.3)

### 2.1 Search strategy

The search was done using the Web of Science, ProQuest, and Science Direct databases. Table 1 lists the keywords and Boolean operators used to find suitable articles. The Web of Science tends to be more generous in its matching, so different search strings were used in the Web of Science.

**Table 1:** Databases and search strings.

**Database**	**Strings for topic and advanced search**
Web of Science	TS=(“meat^*^ consum^*^ reduction” OR “meat intake” OR “meat eating”) AND TS=(“socioeconomic factors” OR “social class” OR “social group” OR “status” OR “social inequality” OR “social stratification” OR “social heterogeneity” OR “social diversity”) Socio-economic factors AND “meat consumption” Social class AND “meat consumption” Reducing meat consumption AND socioeconomic group^*^ Reducing meat consumption AND social class^*^
ProQuest	(“meat consumption reduction”) AND (“socioeconomic factors” OR “social class” OR “social group” OR “status” OR “social inequality” OR “social stratification” OR “social heterogeneity” OR “social diversity”) (“decreasing meat consumption”) AND (“socioeconomic factors” OR “social classes” OR “social groups” OR “status” OR “social inequality” OR “social stratification” OR “social heterogeneity” OR “social diversity”) (“drivers” OR “facilitators” OR “advantages” OR “barriers” OR “challenges”) AND (“to reduce meat consumption” OR “to decrease meat consumption” OR “to lower meat consumption” OR “on the reduction of meat consumption”)
Science Direct	(“meat consumption reduction”) AND (“socioeconomic factors” OR “social class” OR “social group” OR “status” OR “social inequality” OR “social stratification” OR “social heterogeneity” OR “social diversity”) (“decreasing meat consumption”) AND (“socioeconomic factors” OR “social classes” OR “social groups” OR “status” OR “social inequality” OR “social stratification” OR “social heterogeneity” OR “social diversity”) (“drivers” OR “facilitators” OR “advantages” OR “barriers” OR “challenges”) AND (“to reduce meat consumption” OR “to decrease meat consumption” OR “to lower meat consumption” OR “on the reduction of meat consumption”)

### 2.2 Study eligibility criteria

The literature research included peer-reviewed articles with original empirical results published in English between 2019 and 2024, regardless of the discipline. To give an overview of the studies' representativeness and aims, the method applied for the data collection and the sample size are listed in the Supplementary material (see Table 1).

Besides studies that investigated direct results relating to social groups' meat consumption, the review included literature on vegetarianism and veganism, as some of these studies hint at the behavior of specific groups relating to a meat diet. Articles not providing results about specific social groups, class or status were excluded. Moreover, articles about consumers' attitudes toward and views on meat consumption that did not provide information about their habits or interventions relating to meat consumption were excluded. Additionally, articles focusing on the quality of meat and the problems or preferences related to bush and wild meat, hunting, and other specific types of meat were excluded.

Only literature referring to populations in upper middle-income and high-income countries was included because these countries consume the most meat. The definition of middle- and high-income was based on the “New World Bank country classifications by income level: 2022–2023” (World Bank, 2022). Conference papers, dissertations and non-scientific literature, such as newspaper articles, blog posts, and websites, were not included.

### 2.3 Screening of the studies

**Figure 1:**
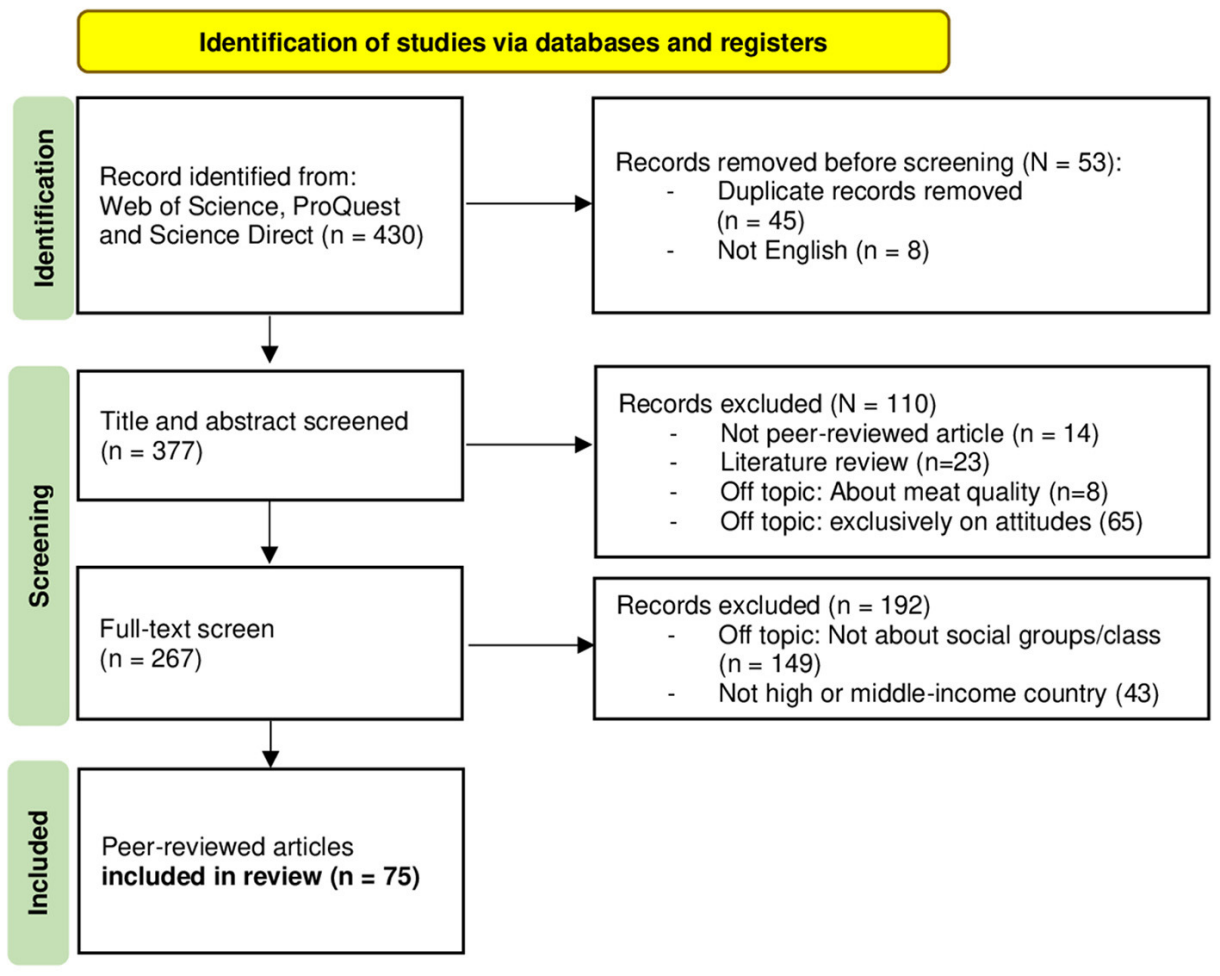
PRISMA flow diagram, source of template: Page et al. (2021).

The studies were screened in two stages: 1) title and abstract screening, to eliminate studies that do not meet inclusion criteria; 2) full-text review to apply the predefined inclusion and exclusion criteria. The results of this selection process are illustrated in the PRISMA flow diagram, showing the number of records identified, screened, included and excluded (see Figure 1).

In the first step, 430 literature sources were collected. After deleting duplicates and literature in languages other than English, 377 entries remained. Excluding 110 items, such as literature that was not peer-reviewed or published in scientific journals, and that did not satisfy the criteria of covering the topic of meat or habits about meat consumption or interventions, 267 entries remained. These entries were fully screened for topic criteria focusing on socio-economic groups, social groups, social class or status, and research applied in high- or middle-income countries, which led to the exclusion of 192 items and, finally, the inclusion of 75 peer-reviewed articles.

A category system was created in the literature management programme Zotero to organize and screen the collected literature. The main categories were created according to the topics specified in the research question. The sub-categories were built according to the found literature. One category included sub-categories on behavior within socio-demographic groups, such as age, gender and family status, and status-related groups, such as income, occupation and education, and another category included sub-categories on social norms and context effects. A third category included sub-categories on the intervention options for the same socio-economic groups, status-related groups and social norms.

## 3 Results

This section presents the findings on the differences in meat consumption between social groups in middle- and high-income countries. Based on the clusters that emerged from the current empirical research reviewed in this paper, the first sub-section contains the findings on meat consumption by gender, age, family status, social status, social norms and context. The second sub-section presents the intervention options for reducing meat consumption based on gender, age, family, social status, norms and context.

### 3.1 Meat consumption of different social groups

#### 3.1.1 Gender

Regarding meat consumption habits by social groups, most of the empirical results relate to gender differences. These research results widely reveal that women consume less meat than men (De Backer et al., 2020; Frehner et al., 2021; Lehto et al., 2022; Rosenfeld and Tomiyama, 2021). In particular, red meat is less often consumed by women than by men (Giacoman et al., 2021; Mesler et al., 2022; Neumann et al., 2024; Peeters et al., 2023; Willits-Smith et al., 2023). Women are also more open to plant-based alternatives (Patinho et al., 2021), tend to eat more fruits and vegetables than meat (Oncini and Triventi, 2021) and are more likely to identify as flexitarian, vegan or vegetarian (Çoker et al., 2024; Rosenfeld et al., 2020; Rosenfeld and Tomiyama, 2021; Tschanz et al., 2022). Women who are concerned about societal standards of attractiveness for women tend to prefer plant-based options, associating them with beauty and health, as Chan and Zlatevska (2019) revealed in three quantitative studies including 268 undergraduates, 878 Americans from Mechanical Turk and 489 Britons from Prolific Academic. Men with lower perceived socio-economic status exhibit a greater preference for meat, as it symbolically represents the status they aspire to achieve (Chan and Zlatevska, 2019).

Men and women, moreover, handle their meat consumption differently: men tend to defend their meat consumption directly. In contrast, women avoid recognizing their responsibility for the source of meat (the fact that animals need to be killed for meat; Mertens and Oberhoff, 2023).

These gender differences are explained by the fact that women, compared to men, on average score higher in nutrition knowledge (Klink et al., 2022) and higher in regard to concerns for animal welfare (Ioannidou et al., 2023) and the environment (da Veiga et al., 2023). Moreover, in many societies, meat is strongly related to masculine values and power (Lax and Mertig, 2020; Randers and Thøgersen, 2023). For example, barbequing reproduces hegemonic masculinity, and meat meals reflect cultural traditions relating to masculinity (Carper, 2020; Carroll et al., 2019). Perceptions of meat are primarily based on individuals' identification with gender (Mertens and Oberhoff, 2023; Peeters et al., 2023). Men in patriarchal and sexist social environments show high tendencies to perceive veganism and vegetarianism as feminine and men who do not eat meat as less masculine, as seen in Carper's (2020) results based on semi-structured interviews with 14 men in Turkey Camilleri et al.'s (2024) quantitative study in Australia and England shows that male participants who were found to support the use of physical violence and to value sexual virility highly ate more meat. On the other hand, men who were willing to reduce their meat consumption had gender egalitarian values.

#### 3.1.2 Age

Regarding age and meat consumption, most studies claim a reversed u-shaped relation (e.g., Faber et al., 2020; Raptou et al., 2024). Younger individuals tend to consume more meat, especially red meat, compared to older individuals, while middle-aged adults eat the most meat in general (Frehner et al., 2021; Koch et al., 2019; Lehto et al., 2022; Tschanz et al., 2022). Elderly individuals tend to stop eating red meat (Giacoman et al., 2021) or eat less red meat (Willits-Smith et al., 2023) and perceive meat as less important (Sares-Jäske et al., 2022).

However, several studies suggest that younger age groups are more open to non-traditional diets. For example, younger age groups were found to perceive alternatives, such as cultivated meat (Szejda et al., 2021) and plant-based meat, more positively (Knaapila et al., 2022). Additionally, Knaapila et al. (2022) argue, based on their quantitative secondary analysis on data from the German National Nutrition Survey II, including 12,733 participants, that millennials have more significant environmental concerns and environmental knowledge than older generations. Raptou et al.'s (2024) quantitative study using cross-sectional data from university students in Greece, India and the UK, including 528 participants born between the mid-to-late 1990s and the early 2010s, supports this. It reveals that health aspects are the main driver for university students' consumption of more plant-based food and less meat. However, the study also demonstrates that this health motivation can lead to more meat consumption due to a desire to increase one's protein intake. Young Indian students who are very committed to sports are less likely, by 27.8%, to move toward more plant-based dietary patterns.

#### 3.1.3 Family status

Family status can influence women's eating behavior and men's perceptions of meat: Sares-Jäske et al. (2022) found in their secondary analysis of the FinHealth Study, with a sample of 4,671 individuals, that women living in households with children consumed more red and processed meat than women without children. However, they did not have more critical attitudes toward meat consumption. On the contrary, men with children had more negative views on meat but did not consume it more than men in households without children.

Hesselberg et al. (2024) found, in a Danish survey, that parents, particularly mothers, were responsible for implementing new green preferences, including meat-reduced dishes, in everyday consumption, indicating a gendered division of labor in meal planning and preparation. Family cohesion and harmony were found to be drivers of and barriers to reducing household meat consumption. Moreover, children played a crucial role as “gatekeepers” in influencing household food consumption patterns in regard to more sustainable diets, often exploring new food options.

#### 3.1.4 Social status

As emphasized in the introduction, several theories suggest that consumption is a significant way of achieving social distinction and acts as a symbol of status. Empirical research broadly shows the effects of education or income on meat consumption. Less research exists on the relationship between meat consumption and different professions. In one exception, Ge et al. (2022) indicate that skilled workers and manual laborers tend to eat more meat than professions such as civil servants, office workers, teachers, etc. Interestingly, Lax and Mertig's (2020) online survey with 584 participants in the United States shows that manual laborers perceive meat as more masculine than persons in other occupations.

Results on the connection between income and meat consumption indicate that in high- and middle-income countries there is mainly a negative relation between higher education and meat consumption (Einhorn, 2021; Franchini et al., 2024; Loginova and Mann, 2024; Mata et al., 2023; Sares-Jäske et al., 2022). Exceptionally, a secondary analysis by Lehto et al. (2022) using the National Finish health databases from 2007, 2012 and 2017, including 4,874, 4,812 and 4,442 participants, does not show any significant relationship between meat consumption and income. Most research indicates that low-income groups usually face more significant barriers to eating plant-based food (e.g., Klink et al., 2022; Kuosmanen et al., 2023; Piracci et al., 2023) and tend to opt more often for cheaper meat products than those in higher-income classes (Klink et al., 2022). The main barriers faced by groups with low income and low education in food access are time constraints, perceived cost, cooking knowledge, taste, and cultural preferences (Ludwig-Borycz et al., 2023). Financial insecurity leads to a lack of time for cooking but also diverts mental resources from food planning and preparation, researching alternative (non-meat) recipes, and interest in increasing culinary knowledge, as Einhorn (2021) explains based on her interviews with 46 Germans aged 19 to 71.

However, in upper middle-income countries, such as China or Brazil, where the economy has grown more recently, meat is still something that only high-income groups can afford. Thus, low-income groups consume less meat. For example, Balcázar's (2020) study surveying 358 meat eaters and non-meat eaters in China demonstrates a direct relationship between income levels and meat consumption, indicating that high-income groups eat more meat due to their higher income. In addition, Giacoman et al.'s (2021) findings based on a survey with 2,017 participants in Chile show that people with higher household incomes are 36% less likely to stop consuming red meat than those from the poorest household income groups measured.

Regarding education, most research indicates that higher-educated individuals tend to eat less meat. Mata et al.'s (2023) results based on two cross-sectional surveys across Europe (*n* = 9,149–10,226) indicate that higher education levels were associated with lower processed meat consumption, and attitudes toward food partially mediated the relationship between education and consumption. They explain this by the attitudes of more highly educated people toward healthier diets (Mata et al., 2023). Another explanation is that environmentally conscious consumers (Pais et al., 2023) and individuals who are interested in nature conservation are more likely to make sustainable food choices, such as reducing meat consumption (Haider et al., 2022). Pais et al. (2023) revealed in a quantitative study in Portugal that the more environmentally conscious and informed the consumers are, the more likely they are to choose more plant-based and less animal-based meals every week.

Lehto et al. (2022) showed that the middle and high-education groups were more likely to be low meat consumers. Also, higher-educated British meat eaters were associated with lower meat consumption norms in an online cross-sectional survey, including 398 meat lovers, 103 heavy meat consumers, 158 flexitarians and 546 moderates in a study by Wolfswinkel et al. (2024). In Italy, Oncini and Triventi (2021) found in a survey that highly educated individuals were more likely to eat vegetables and fruits than meat. Ludwig-Borycz et al.'s (2023) analysis of second data analysis in the United States, of 1,308 young participants, provides evidence that overconsumption of meat was lower when the participants were highly educated. In Germany, Klink et al. (2022) found in their quantitative study that individuals with higher educational attainment engaged in more sustainable and health-conscious dietary behaviors, meaning consuming less animal-based food. Giacoman et al. (2021) revealed that postgraduates were twice as likely to stop eating red meat for environmental reasons than those with primary education. In Switzerland, Frehner et al. (2021) found that beef consumption was lower than chicken consumption among participants with a tertiary education. Loginova and Mann's (2024) findings on secondary data from the Swiss Governmental Statistics, including 62,871 observations, confirmed that meat was consumed more among lower-education groups in Switzerland, although the higher-educated households might eat more fish than other meat products.

Very few empirical studies have been conducted on social status or social class as a concept connected to meat consumption. The existing studies in this literature review measured social status by combinations of education, profession, and income, commonly used proxies for measuring social status. Empirical research supports this approach, regarding meat consumption. Chan and Zlatevska (2019) found in experiments in Canada, the United States, and the Netherlands that individuals who assessed their social status as low preferred meat more than those with high self-assessed status. The authors interpreted this as meaning that individuals see meat as substitutable for the status they lack.

In addition, a quantitative study by Giacoman et al. (2021) in Chile underlines the connection of meat consumption to social status, particularly the Weberian notion of social class. The authors used education, types of profession classes (casual and informal jobs, unskilled laborers, and domestic service), and household income as indicators to measure social class. The upper class was more likely to stop consuming red meat for environmental reasons than the working class (Giacoman et al., 2021). However, middle class people were less likely than working-class people to stop consuming red meat (Giacoman et al., 2021). The authors interpret that the middle class still desires red meat, as it was inaccessible in the past, while the upper class no longer does due to bad health and environmental effects (Giacoman et al., 2021). For the working class, red meat is appreciated but too expensive (Giacoman et al., 2021). A qualitative study with biographical interviews with 40 young vegans in Chile by Giacoman and Joustra (2024) highlights the significant role of social class in shaping vegan practices. They define social class by a combination of education, income, profession, and the area where the individuals live. The key findings of the qualitative research reveal that individuals from upper social classes are inclined to healthy foods from different cultures, which facilitates their openness to new flavors and reflective eating habits, aiding their transition to veganism. However, individuals from lower social classes also show prerequisites in favor of veganism: they practice traditional meatless cooking, mainly due to budget constraints, which makes the shift to a plant-based diet more accessible to them.

Einhorn (2021) conducted a qualitative study in Germany on meat consumption and social class. She defined social classes by education and income, which revealed slightly different results. Her results similarly showed that the higher social classes consumed less meat and considered environmental and animal welfare more important than the lower classes. Nevertheless, the lower social classes often viewed meat consumption as essential for a “proper” diet, influenced by material and cultural resources.

Vos et al. (2022) defined social status in their qualitative research in Belgium by educational level, occupational status, and income. Parents with higher social status applied sustainable food choices but found meals with less meat more challenging. Low self-efficacy relating to sustainability was found to be the reason for this. Groups with lower social status stated the same thing, but had different reasons. Their reasons were mainly high prices and a lack of inspiration and skills, which hindered choosing sustainable and healthy food.

Markoni et al. (2023) compare the social status influences on meat consumption in Vietnam and Switzerland using qualitative methods and find that meat consumption is connected to notions of prosperity and social status in both countries. In Vietnamese culture, serving meat at meals often symbolizes wealth and success. The ability to afford meat reflects a higher socio-economic status, making it a status symbol in social settings. Traditional values reinforce the role of meat as central to hospitality and celebration, further linking it with prestige. In Switzerland, status is less tied to the quantity of meat consumed. Instead, choosing sustainable, high-quality and organic meat symbolizes higher cultural capital in Swiss society.

#### 3.1.5 Social norms

Previous research on dietary practices in general has shown that cultural norms and values significantly guide these practices (e.g., Paddock, 2017). Oleschuk et al. (2019) conducted semi-structured interviews with 77 Canadians and showed that their meat choices were influenced by broader societal expectations and group identities, reflecting the interplay between individual agency and cultural context.

Lund and Halkier (2024) demonstrated the potential of dynamic norm interventions in an experimental study in the United Kingdom. Social norm messages, such as “More and more customers are choosing vegetarian options,” displayed on digital menu screens led to a significant increase in sales of plant-based meals.

Hielkema and Lund's (2021) survey with 1,005 participants in Denmark revealed that social group influence and cultural perceptions are important factors shaping plant-based meat choices. Participants in their study who had friends or family who reduced or avoided meat were observed to be more likely to change their meat consumption.

Horgan et al.'s (2019) results from their analysis of secondary data of 4,156 participants in the United Kingdom show that people are more likely to eat meat in the company of others, particularly family and friends. Moreover, many people tend to cook more meat when hosting guests, responding to perceived social expectations (Biermann and Rau, 2020). Eating at friends' and family members' homes positively correlates with meat consumption in Switzerland and France but not in the Netherlands (Laffan, 2024). Additionally, Markoni et al. (2023) found by comparing group discussions in Switzerland that meat is part of the meals consumed on special occasions and in social gatherings.

Grünhage and Reuter's (2021) results of a survey with 670 participants in Germany suggest that dietary choices are intertwined with broader political, social and moral values. Accordingly, individuals with left-leaning or centrist political attitudes are more likely to adopt veganism or vegetarianism due to their more substantial moral commitment to avoiding harm and ensuring fairness, particularly regarding animal welfare and environmental concerns. Stanley (2022) found in her quantitative study with 197 individuals in Australia and 453 in the United States that symbolic concerns, such as threats to national dietary customs and cultural identity, are the primary drivers of negative attitudes toward vegetarianism, rather than economic fears.

According to other studies, cultural norms regarding meat consumption can also be reflected in ethnic differences. For example, Çoker et al. (2024) found ethnic differences in the UK between South Asian, White and Black British respondents by applying an online survey including 402 White, 382 South Asian and 229 Black people. South Asian respondents were significantly less likely to eat meat than White respondents. The authors found no significant difference between White and Black respondents. South Asian and Black respondents reported being more influenced by friends and family in their food choices and eating similarly to their friends and family, compared to White respondents.

#### 3.1.6 Context effects

Research additionally indicates that a person's location and its context affect individuals' meat consumption habits. A quantitative study by Laffan (2024) in the Netherlands, France and Switzerland shows that people tend to eat more meat when dining out, particularly in restaurants and cafes. This could be due to the enticing meat options available at such establishments or the perception of eating out as a special occasion for indulging in meat.

Also, Ritzel and Mann's (2023) analysis of the US National Health and Nutrition Examination Survey data, with 41,262 observations, shows that frequent eating out is associated with higher meat consumption (Ritzel and Mann, 2023). However, not all social groups consume more meat when eating out, as one affluent group showed no significant increase in meat intake compared to those eating primarily at home (Ritzel and Mann, 2023). Biermann and Rau (2020) confirmed these differences between dietary types regarding context with their survey of 420 responses in Germany: omnivores often preferred eating meat in restaurants rather than at home, while flexitarians were more likely to reserve meat consumption to when they dined out.

However, according to Wolfson, Willits-Smith, Leung, Heller and Rose (2022) secondary analysis of the National Health and Nutrition Examination Survey 2007–2010 (*n* = 11,469) in the United States, individuals cooking at home have higher carbon footprints than those consuming takeout or fast food. This is attributed to the tendency to prepare more meat-intensive meals when cooking at home, especially beef (Wolfson et al., 2022). On the other hand, where workplaces have limited plant-based alternatives, this leads to higher meat consumption than home settings, where time and convenience can push individuals toward non-meat options, as Pluck and Morrison-Saunders (2022) showed with semi-structured interviews with 33 employees of a financial services consultancy in London.

### 3.2 Interventions to reduce meat consumption

In many of the studies in this literature review, the authors derived recommendations from findings about specific social groups' meat consumption habits. Some of them tested interventions, mainly nudging interventions. However, the state of the art does not yet provide empirically tested measures for all of the social groups described in this literature review.

As most research focuses on gender-specific meat consumption, many recommendations focus on interventions to reduce males' higher meat consumption. Pohlmann (2022) tested the food choices of men in four experimental studies. To challenge the link between meat consumption and masculinity, Pohlmann (2022) suggests that moral appeals are effective for men. By reframing meat reduction as a compassionate, strong and ethical choice, these appeals aim to reduce the perceived threat to masculinity that some men feel when considering plant-based diets. For resistant male consumers who eat the most meat, interventions that focus on shifting the cultural association between meat consumption and masculinity can challenge the idea that “real men” must eat meat. For ambivalent male consumers who eat moderate to high amounts of meat and show some willingness to reduce meat consumption, health-based and environmental appeals could be effective, as they may already be questioning their meat intake but need stronger motivations to act. For males who already eat minimal meat and are open to further reduction, the authors suggest messages around ethical and environmental benefits, further reinforcing their attitudes (Pohlmann, 2022). Camilleri et al. (2024) recommend, based on their cross-sectional survey results with 557 Australian and English males, that public health and environmental policymakers consider the psychological and cultural drivers of food choices to develop more effective interventions. The authors suggest focusing on dissociating meat from high status and masculinity, highlighting plant-based diets' health and status benefits, and applying tailored approaches by addressing distinct subgroups based on their psychosocial characteristics.

Raptou et al. (2024) refer to younger generations as a crucial group for meat reduction interventions, not because of their high levels of meat consumption but in order to meet future consumer needs and foster sustainable, long-lasting and environmentally friendly choices. The authors found, from their cross-sectional data analysis including 528 participants, that providing information on plant-based foods can help boost familiarity with, and reduce neophobia toward, these products among young groups. Frehner et al. (2021) also see young groups as offering potential for policies to reduce a population's total impact. The authors see young groups as potential role models for sustainable nutrition. To meet young adults' protein and nutrient needs, Raptou et al. (2024) suggest that the food industry should invest in fortifying plant-based foods to ensure a balanced diet that includes proteins, iron and B12. Knaapila et al. (2022) see the potential to reduce meat consumption in young groups that neither strongly prefer nor strongly resist meat intake. These young groups with a middle preference for meat are perhaps the best targets for policy initiatives promoting meat reduction through plant-based alternatives. Koch et al.'s (2019) results, based on a secondary analysis of the German National Nutrition Survey II, including 12,733 participants, indicate that young adults who consume a lot of meat may find reducing portions within their preferred meal structure easier than adopting entirely new vegetarian meals. In any case, meat-reduced or vegetarian meals must, in the authors' view, be appealing, easy to prepare, and fit well into current habits to be accepted as a genuine alternative.

Hesselberg et al. (2024) see, based on their results from interviews with 19 mothers, 11 fathers and 26 adolescents, the potential for interventions to reduce meat consumption in family and parenting situations. The authors emphasize the importance of negotiations, relations and emotional aspects of family life in household dietary choices. To achieve sustainable meat consumption, the family, as a collective consumer unit, is important. Collaborative activities such as shared meals, cooking and exchanging ideas resonated with participants in the authors' study, suggesting a promising avenue for future sustainable eating initiatives. Rather than relying on individualistic approaches to public policy and interventions, emphasizing the social dimensions of food could, according to Hesselberg et al. (2024), enhance the effectiveness of campaigns promoting sustainable eating. Moreover, interventions targeting specific family members, such as fathers, could facilitate change by redistributing food-related responsibilities and introducing new skills and interests that encourage healthier and more sustainable eating practices (Hesselberg et al., 2024). Finally, the study highlights the potential of older children in regard to promoting sustainable eating behaviors as initiators or “gatekeepers” in their households. School-based food programmes were found to be a vital source of inspiration for younger participants, supporting the role of educational institutions in fostering environmental awareness and encouraging green dietary habits (Hesselberg et al., 2024).

As lower-education groups were found to consume higher amounts of meat than higher-education groups, research recommends several kinds of education and information interventions. Klink et al. (2022) suggest that future efforts should be directed toward education interventions relating to nutrition and the interpretation of food labels to compensate for differences in dietary behavior among groups with different levels of education. Training in plant-based cooking and increased confidence in cooking at home could, according to Biermann and Rau (2020), support a transition to sustainable food practices, with additional policy tools, like subsidies and store-level interventions, promoting these changes. Other authors of the screened studies conclude that information about the harmful effects of meat consumption, and the health and environmental benefits of plant-based nutrition and the preparation of tasteful plant-based meals, is crucial to reduce meat consumption (Craig et al., 2021; Faber et al., 2020; Fehér et al., 2020; Fesenfeld et al., 2023; Perino and Schwirplies, 2022).

As the level of meat consumption differs significantly between income groups, several authors recommend implementing a pricing policy, particularly taxes on meat products, as an effective tool for reducing meat consumption (Bielik et al., 2021; Chen et al., 2024; Pechey et al., 2022; Pereira et al., 2023; Pitt et al., 2020). However, considering the evidence showing that in high-income countries, lower-income groups eat more meat than the high-income groups, the high prices of vegetables and other plant-based alternatives are barriers to reducing meat consumption. For example, students in Greece, India and the UK perceived the prices of plant-based food as an obstacle to reducing meat consumption (Raptou et al., 2024). Thus, several authors suggest lowering the costs of plant-based alternatives while increasing meat prices (Broeks et al., 2020; James et al., 2022; Newton and Blaustein-Rejto, 2021; van den Berg et al., 2022).

As social norms and interactions have been shown to influence meat consumption, research also provides recommendations for making it more socially acceptable to reduce one's meat intake in specific settings (Wendler, 2023). Individuals who are more open to social influence and those who do not reinforce their meat-eating behaviors are more likely to change their dietary habits. Ge et al. (2022) showed this in a quantitative study in England. Flexitarians are particularly affected by their social networks and are willing to change their meat-free or meat consumption status.

At celebratory gatherings where meat is expected, gradual introductions of meat-like alternatives and smaller portions may help ease the transition to less meat-heavy menus (Markoni et al., 2023). Markoni et al. (2023), based on results from six online group discussions with 42 Swiss and 44 Vietnamese participants, underline the importance of social interaction by highlighting the importance of community-based projects and infrastructure in living areas, enabling, for example, urban farming. The authors believe both actions can enhance trust between consumers and producers and promote alternatives to mass-produced meat.

However, the social facilitation effect has been shown to be particularly substantial in leisure settings like restaurants, where the probability of consuming meat increases (Wendler, 2023). Çoker et al. (2024) investigated whether dynamic social norm messages could reduce meat consumption in 22 retail store restaurants using a randomized cross-over trial. While the intervention increased awareness of changing dietary trends, it did not significantly reduce meat consumption among participants. Horgan et al. (2019) showed that dining settings in social companies can nudge individuals toward vegetarian options, particularly when shared plant-based dishes are available, and price incentives for group vegetarian meals in restaurants or supermarkets can reinforce this shift. Increased vegetarian options in restaurants, facilitated through choice architecture like nudging, could also significantly impact food sustainability (Markoni et al., 2023). Biermann and Rau (2020) emphasize that organizations promoting plant-based diets should work together to elevate the topic beyond private spaces, with chefs able to play a key role in redefining meat's place on the menu.

In addition, workplace interventions can also foster lasting dietary changes. Horgan et al. (2019) demonstrated the impact of norm-based messaging on food choices in a workplace cafeteria. A poster stating that most people include vegetables in their meals led to a rise in vegetable-based meal selections compared to the baseline period. This influence on purchasing behavior persisted after the poster was removed (Horgan et al., 2019). Moreover, limiting the availability of meat products in this environment could help establish norms among co-workers that promote reduced meat intake, potentially leading to lasting changes in eating habits in both professional and social settings. Moreover, public institutions such as schools are seen, in research, as promising contexts for inventions to reduce meat consumption. Markoni et al. (2023) suggest that cafeterias could offer meals with less or no meat, supported by training in vegetarian cooking and necessary infrastructure. Such initiatives might reshape children's food preferences, leading to “substituting practices” at home (Markoni et al., 2023).

Laffan (2024), using secondary data from the national nutrition surveys of France (*n* = 25,595), Switzerland (*n* = 19,544) and the Netherlands (*n* = 26,683), and Wolfson et al. (2022), recommend emphasizing plant-based eating as a widespread or desirable behavior in public messages, as they believe this can influence choices in various settings, including restaurants and workplaces. The approaches of social marketing campaigns, nudges and messaging highlight the interplay between individual choices and social influences in transitioning toward more sustainable diets.

## 4 Discussion

This review of levels of meat consumption among social groups has revealed that differences are mostly based on gender, social status, and class, including specific factors such as education and income, family status and social norms.

These results align with Stoll-Kleemann and Schmidt's (2017) review: the link between higher education levels and younger age and lower meat consumption, and between men and higher meat consumption, has not changed.

The results on the different consumption behaviors of different groups suggest that effective measures to reduce meat consumption should address the specific needs of the various social groups. According to this review's results, groups that should be especially motivated and supported in reducing their meat consumption are males with strong identification with masculinity, individuals with right-wing political attitudes, middle-aged groups, middle and low classes, middle- and low-income groups, lower educated groups, individuals working as laborers, and sport-oriented groups, as they eat the highest amounts of meat.

For several norm- and value-driven groups, such as political groups and male groups with strong masculine gender identities that highly value meat as a status symbol, similar measures might be successful. The values of these groups, such as conservatism and masculinity, are connected and overlapping. To reach these groups, plant-based meals should be promoted as desirable, delicious, joyful and suitable for all social settings, from everyday meals to festive occasions (Hoek et al., 2017; Stoll-Kleemann and Schmidt, 2017; Taufik et al., 2019). Ecological and ethical reasons might be less convincing for people with conservative and traditional attitudes. Positive attitudes toward meat reduction rather than perceived social pressure might prove more effective (Krispenz and Bertrams, 2020). This indicates the complexity of measures for reducing meat consumption: values cannot be changed solely by means of environmental education and countering the values of these groups. Values and norms are often connected and overlapping, and their roots and reasons are complex and, accordingly, difficult to address.

Information about the health benefits of plant-based food might effectively address groups that are especially interested in health, such as sport-oriented groups that need higher amounts of protein. Shifts in the weighting of plant-based foods more than animal products could contribute to changing perceptions of healthy diets. New food pyramids, as recently published, e.g., in Germany (BZFE, 2024), indicate that plant-based alternatives, such as lentils, can cover the demand for protein.

Education about cooking diverse plant-based meals and their health and environmental advantages (Graça et al., 2019), such as cooking courses in schools, might affect the consumption of these specific social groups. Providing information on the benefits of plant-based meals and plant-based cooking classes in school is also an essential measure for low-education groups. Additionally, labels on meat products were found to be an effective way to inform consumers generally about their environmental impacts (Lohmann et al., 2022; Potter et al., 2023) and were the most accepted measure in the UK, followed by media campaigns, reduced availability, and incentives (Pechey et al., 2022). However, information should be specifically targeted to meet different social groups' interests (Garnett et al., 2015; Joyce et al., 2012), and more knowledge is still needed to achieve this.

Most research recommending economic measurements to shift meat consumption patterns focuses on taxation on high-emission meat products. However, research shows that pricing policies can disproportionately affect low-income groups (Levasseur et al., 2024; Pechey et al., 2022). Measures to reduce meat and enhance sustainable nutrition potentially affect societal groups unequally (Frehner et al., 2021). Under-privileged individuals (e.g., those who work as laborers, with lower education and experiencing financial insecurity) who eat less meat have a higher risk of being overweight than more privileged individuals (Levasseur et al., 2024). They tend to replace meat with energy-dense foods and beverages, including ultra-processed foods, while more privileged individuals have better opportunities to replace meat with healthy alternatives (Levasseur et al., 2024). Thus, it is essential to ensure that plant-based options are affordable and accessible for these less privileged groups (Springmann et al., 2018). Meat taxation could be utilized to subsidize plant-based food (Broeks et al., 2020; Newton and Blaustein-Rejto, 2021).

Generally, it can be summarized that food offers and individuals' capability to change dietary behavior are significant factors in reducing meat consumption across cultures and countries (da Veiga et al., 2023). Many socio-economic categories are linked to and influence each other and have to be seen within the complex context influencing possibilities of behavior. For example, income and education mostly correlate. Several authors recommend, regardless of the consumer's social group, improving the visibility, accessibility and convenience of meat alternatives in public places, such as work, schools and hospitals (Rosi et al., 2022; Vandenbroele et al., 2021; Venema and Jensen, 2024). Setting plant-based options as the default across the different life contexts of individuals is essential to reduce meat consumption (Prusaczyk et al., 2021). When plant-based options are more visible and accessible, such as in worksite cafeterias or public institutions, meat consumption tends to decrease (Reinders et al., 2017; Venema and Jensen, 2024). This can be done by listing alternative plant-based meat dishes as the menu's first and most frequently offered dishes (Taufik et al., 2019), by changing the ratio between meat and vegetables in a dish so that more vegetables are served than meat, focusing more on taste and presenting vegetables (Reinders et al., 2017), or by enlarging the offer of plant-based meals (Rosi et al., 2022). Offering more plant-based meals in canteens can lead to reduced meat consumption at home (Verfuerth et al., 2021).

Additionally, experience with meat substitute products can reduce the intention to eat meat products, as Fesenfeld et al.'s (2023) results from an online survey with 2,590 citizens from China and the United States indicate. One option with high potential might be offering *in-vitro* meat (Bryant et al., 2023). However, *in-vitro* meat is not yet widely accepted by most people and is still very expensive (Bryant et al., 2023; Laestadius et al., 2014). Meat substitutes are often overprocessed (Jahn et al., 2021). Overcoming these obstacles through technical innovation is needed, as is the provision of information about the health and sustainability benefits of these products (Fesenfeld et al., 2023; Jahn et al., 2021).

These technical innovations and other measurements, such as tax policies, financial subsidies for plant-based food, and education and information campaigns, depend on political will and societies that vote for political parties engaged in a social-ecological and just food transformation. Stoll-Kleemann and Schmidt (2017) already concluded, based on their review in 2017, that a lack of policies promoting reduced meat consumption and subsidies for meat production inhibits significant change. Awareness campaigns and the availability of meat alternatives are helpful but insufficient to drive substantial reductions in meat consumption.

Ultimately, this literature review has to be seen in the light of its limitations. First, the lack of a formal bias assessment is a limitation. Without a structured evaluation of study quality, the influence of potential biases, such as selection or reporting bias, cannot be fully determined. Future systematic reviews on this topic should incorporate a formal risk-of-bias assessment using standardized tools, such as the Cochrane Risk of Bias Tool or ROBINS-I, to enhance methodological rigor.

Second, the review's time frame and geographical scope were limited. Due to the focus on literature on high- and middle-income countries, this literature review tends to have a Western and Eurocentric perspective on meat consumption. Expanding the geographical scope could provide deeper insights into how cultural, political and environmental factors shape meat consumption. A wider literature review including all income countries, and thus more Global South countries, could reveal cultural and postcolonial effects on meat consumption. Moreover, very little empirical literature is published in English on the question of how the colonial history has influenced meat consumption in Global South countries and other postcolonial countries.

Third, the review included only literature written in English. Thus, findings published in other languages are missing. A future literature review could provide valuable insights by adding literature published in languages other than English and including search websites such as Scielo and academia.edu. In particular, including Spanish and Portuguese literature would widen the scope of a review on this topic. In South and Middle American countries, such as Brazil, Argentina and Mexico, as well as European countries, such as Spain and Portugal, between 200 and 316 grams of meat are consumed per day and per capita, which is in the top rank, globally. Significant literature has been published in Spanish (e.g., Espejo et al., 2025; Zuazo and Amarista, 2023) and Portuguese (e.g., Barros, 2024; Bertoncelo, 2019; Groot, 2020) and would give additional insights on the topic of meat and social groups. Moreover, including literature written in Arabic would give more insights into high meat consumption countries, such as Saudi Arabia.

Due to the current state of the art, this review varies in the amount of information it provides, depending on the social group or class, both in terms of behavior and intervention options. This indicates research gaps in the field. Future empirical research investigating the factors influencing social groups should delve deeper into the complex interplay between social class and meat consumption, as, for example, introduced in this article by Bourdieu's concept of cultural, social and economic capital. Consumers with lower social status are affected by more complex and indirect barriers to reducing meat consumption than just low income or low education levels. For example, lower self-efficacy among groups with lower social status might lead to lower capabilities to change diet habits toward unconventional diets, such as reduced meat consumption (e.g., Einhorn, 2021). In contrast to individuals with higher status who want to distinguish themselves from others, individuals with lower status tend to stick to the diet that is predominant in their social networks, which, in Western cultures, mainly includes meat, because they are more dependent on their social network. Moreover, social groups with higher educational and income levels have the mental, financial and time resources to pursue an interest in food generally, in unconventional meals, such as dishes from other cultures, and vegetarian or vegan dishes, and to research, taste and cook different foods (Einhorn, 2021; Raptou et al., 2024). Evidence on these theoretical assumptions could contribute to developing measures to support sustainable and healthy diets among less privileged societies.

Future research could also shed light on the interactions and influences of different socio-economic factors on each other. For example, it remains unclear if and how much educational factors influencing meat consumption are moderated or mediated by factors of income and the other way around.

The effectiveness of pricing policies, educational campaigns, and policy measures on sustainable meat consumption for specific social groups could also be investigated further. Moreover, cross-cultural research would give insights into the influence of different cultural backgrounds and values on changes in meat consumption and responses to interventions.

Last but not least, practical initiatives are needed to facilitate readily available, affordable, healthy and sustainable food for all social groups so that a diet with sustainable meat levels becomes the norm. For this to happen, policymakers in middle- and high-income countries must take responsibility. Reducing meat consumption and sustainable food should not remain a symbol of status and class distinction but should be equally accessible to all.

## Footnotes

^1^This insufficiency does not apply to all areas globally. Keeping livestock in mountain areas or deserts can be crucial to preserving cultural landscapes and providing humans with nutrition.

## Supplementary material

Supplementary Table 1 Empirical Studies Included in the Literature Review (Section 3 Results).
