# Supplementary material for: Meat consumption among different social groups and specific options for reducing it: a literature review of empirical research
Source: Front Sociol. 2025 May 23;10:1547663. doi: 10.3389/fsoc.2025.1547663 (PMC12141276; doi:10.3389/fsoc.2025.1547663)
Supplement: Supplementary Table 1 — Empirical Studies Included in the Literature Review (Section 3 Results). [file Table_1.docx]

Supplementary Material

# Supplementary Table 1: Empirical Studies Included in the Literature Review (Section 3 Results)

| **Source** | **Data Collection** | **Country** | **Sample Size** | **Sub-samples** |
| --- | --- | --- | --- | --- |
| Bielik, Peter; Hanova, Martina & Benda-Prokeinova, Renate. 2021. "How to be sustainable in beef consumption – Exploring the factors. Case study". AGRICULTURAL ECONOMICS-ZEMEDELSKA EKONOMIKA 67:382–390. doi: 10.17221/80/2021-AGRICECON. | Case study | Slovakia | N/A | N/A |
| Biermann, Gesa & Rau, Henrike. 2020. "The meaning of meat: (Un)sustainable eating practices at home and out of  home". APPETITE 153:1-11. doi: 10.1016/j.appet.2020.104730. | Survey | Germany | 420 | 83.2% omnivores  11.6% flexitarians  1.5% pescatarians  3.4% vegetarians  0.3% vegans |
| Broeks, Marlin J.; Biesbroek, Sander; Over, Eelco A. B.; van Gils, Paul F.; Toxopeus, Ido; Beukers, Marja H. & Temme, Elisabeth H. M. 2020. "A social cost-benefit analysis of meat  taxation and a fruit and vegetables subsidy  for a healthy and sustainable food  consumption in the Netherlands". BMC PUBLIC HEALTH 20(643):1-12. doi: 10.1186/s12889-020-08590-z. | Secondary data analysis | The Netherlands | N/A | N/A |
| Camilleri, Lauren; Kirkovski, Melissa; Scarfo, Jessica; Jago, Andrew & Gill, Peter Richard. 2024. "Understanding the Meat-Masculinity Link: Traditional and Non-Traditional Masculine Norms Predicting Men’s Meat Consumption". ECOLOGY OF FOOD AND NUTRITION 63(4):355-386, doi: 10.1080/03670244.2024.2361818. | Survey | Australia (56.2%), UK (43.8%) | 557 men | 8.3% meat-avoiders  32.5% meat reducers  59.2% unrestricted meat-eaters |
| Çarpar, Mehmet Can. 2020. „Nutrition, Identity and Masculinity: The Sociology of Meat Eating“. ISTANBUL UNIVERSITESI SOSYOLOJI DERGISI 40(1):249-277. doi: 10.26650/SJ.2020.40.1.0041. | Semi-structured interviews | Turkey | 14 men | - |
| Carroll, Julie-Anne, Capel, Eleanor M. & Gallegos, Danielle. 2019. „Meat, Masculinity, and Health for the ‚Typical Aussie Bloke‘: A Social Constructivist Analysis of Class, Gender, and Consumption“. AMERICAN JOURNAL OF MENS HEALTH 13(6):1-12. doi: 10.1177/1557988319885561. | Focus groups and semi-structured interviews | USA | 20 men | - |
| Chan, Eugene Y. & Zlatevska, Natalina. 2019. „Is meat sexy? Meat preference as a function of the sexual motivation system“. FOOD QUALITY AND PREFERENCE 74:78-87. doi: 10.1016/j.foodqual.2019.01.008. | 3 surveys | USA, UK | Study 1:  268 undergraduates  Study 2:  878 Americans from Mechanical Turk  Study 3:  489 Britons from Prolific Academic | Study 1:  52.2% women  47.8% men  Study 2:  42.3% women  57.7% men  Study 3: 64.0% women  36.0% men |
| Chen, Jiao; Sun, Dingqiang; Zhong, Funing; Ren, Yanjun & Li, Lei. 2024. „Can We Design Food Taxes to Reduce Agricultural Greenhouse Gas Emissions in China? A Perspective from Animal Protein Intake of Low-Income Populations“. CHINA AGRICULTURAL ECONOMIC REVIEW 16(3):389-420. doi: 10.1108/CAER-05-2023-0130. | Modelling | China | Low-income populations | N/A |
| Çoker, Elif Naz; Pechey, Rachel & Jebb, Susan A. 2024. „Ethnic differences in meat consumption attitudes, norms and behaviors: A survey of White, South Asian and Black ethnic groups in the UK“. APPETITE 198:1-10. doi: 10.1016/j.appet.2024.107359. | Survey | UK | 1,014 | 39.7% White 37.7% South Asian 22.6% Black |
| Çoker, E. N., Pechey, R., Frie, K., Jebb, S. A., Stewart, C., Higgs, S., & Cook, B. (2022). A dynamic social norm messaging intervention to reduce meat consumption: A randomized cross-over trial in retail store restaurants. Appetite, 169, 105824. | Focus groups | UK | 28 | - |
| Craig, Winston J., Ann Reed Mangels, Ujué Fresán, Kate Marsh, Fayth L. Miles, Angela V. Saunders, Ella H. Haddad, Celine E. Heskey, Patricia Johnston, und Enette Larson-Meyer. 2021. „The safe and effective use of plant-based diets with guidelines for health professionals“. Nutrients 13(11):4144. | Review article | N/A | N/A | N/A |
| da Veiga, Claudimar Pereira, Mirian Natali Blezins Moreira, Cassia Rita Pereira da Veiga, Alceu Souza, und Zhaohui Su. 2023. „Consumer Behavior Concerning Meat Consumption: Evidence from Brazil“. FOODS 12(1):188. doi: 10.3390/foods12010188. | Semi-structured interviews | Brazil | 18 | - |
| De Backer, Charlotte, Sara Erreygers, Charlotte De Cort, Frederic Vandermoere, Alexander Dhoest, Jules Vrinten, und Sofie Van Bauwel. 2020. „Meat and masculinities. Can differences in masculinity predict meat consumption, intentions to reduce meat and attitudes towards vegetarians?“ Appetite 147:104559. doi: 10.1016/j.appet.2019.104559. | Survey | Belgium | 309 meat-eating men | - |
| Einhorn, Laura. 2021. „Meat consumption, classed?“ Österreichische Zeitschrift für Soziologie 46(2):125–46. doi: https://doi.org/10.1007/s11614-021-00452-1. | Semi-structured interviews | Germany | 46 | 23 meat-reduced to heavy meat-diets  23 meat-free |
| Faber, Ilona, Nuria A. Castellanos-Feijoó, Linde Van de Sompel, Aleksandra Davydova, und Federico J. A. Perez-Cueto. 2020. „Attitudes and knowledge towards plant-based diets of young adults across four European countries. Exploratory survey“. Appetite 145:104498. doi: 10.1016/j.appet.2019.104498. | Survey | Belgium (25.1%), Denmark (27.2%), Spain (21.2%),  The Netherlands (26.5%) | 438  aged 18-30 years | 65.1% omnivores  22.6% flexitarians  7.5% vegetarians  3.0% plantarians  1.8% vegans |
| Fehér, András, Micha\l Gazdecki, Miklós Véha, Márk Szakály, und Zoltán Szakály. 2020. „A Comprehensive Review of the Benefits of and the Barriers to the Switch to a Plant-Based Diet“. Sustainability 12(10). | Literature review | Hungary (13.9%), international (86.1%) | 101 literature sources (with both primary and secondary data) | 81 academic articles  16 books  4 other sources from the internet |
| Fesenfeld, Lukas Paul, Maiken Maier, Nicoletta Brazzola, Niklas Stolz, Yixian Sun, und Aya Kachi. 2023. „How information, social norms, and experience with novel meat substitutes can create positive political feedback and demand-side policy change“. Food Policy 117:102445. | Survey | China (47.5%), USA (52.5%) | 2,590 | - |
| Franchini, Cinzia, Beatrice Biasini, Giovanni Sogari, Rungsaran Wongprawmas, Giulia Andreani, Irina Dolgopolova, Miguel I. Gómez, Jutta Roosen, Davide Menozzi, Cristina Mora, Francesca Scazzina, und Alice Rosi. 2024. „Adherence to the Mediterranean Diet and its association with sustainable dietary behaviors, sociodemographic factors, and lifestyle: a cross-sectional study in US University students“. Nutrition Journal 23(1):56. doi: 10.1186/s12937-024-00962-0. | Survey | USA | 1,485 university students  aged 18-24 years | - |
| Frehner, A., H. H. E. Van Zanten, C. Schader, I. J. M. De Boer, G. Pestoni, S. Rohrmann, und A. Muller. 2021. „How Food Choices Link Sociodemographic and Lifestyle Factors with Sustainability Impacts“. JOURNAL OF CLEANER PRODUCTION 300:126896. doi: 10.1016/j.jclepro.2021.126896. | Secondary data analysis (menuCH) | Switzerland | 2,057  aged 18-75 years | - |
| Ge, Jiaqi, Andrea Scalco, und Tony Craig. 2022. „Social Influence and Meat-Eating Behaviour“. Sustainability 14(13):7935. doi: 10.3390/su14137935. | Secondary data analysis (British Social Attitude Survey) | UK | 2,187 | - |
| Giacoman, Claudia, Pamela Ayala Arancibia, und Juan Alfaro. 2021. „Choosing to Stop Consuming Meat for Environmental Reasons: Exploring the Influence of Gender and Social Status Variables in Chile“. BRITISH FOOD JOURNAL 123(9):2996–3013. doi: 10.1108/BFJ-03-2021-0239. | Secondary data analysis (Encuesta Nacional Bicentenario) | Chile | 2,017 | 51.8% women  48.2% men |
| Giacoman, Claudia, und Camila Joustra. 2024. „Veganism, Cuisine, and Class: Exploring Taste as a Facilitator in Adopting a Vegan Lifestyle in Santiago, Chile“. Frontiers in Sociology 9. doi: 10.3389/fsoc.2024.1356457. | Biographical interviews | Chile | 40 vegans  aged 19-35 years | - |
| Grünhage, Thomas, und Martin Reuter. 2021. „What Makes Diets Political? Moral Foundations and the Left-Wing-Vegan Connection“. Social Justice Research 34(1):18–52. Doi: 10.1007/s11211-020-00361-w. | Survey | Germany | 670 | 65.1% omnivores  9.6% pescetarians  19.0% vegetarians  6.4% vegans |
| Haider, Verena, Franz Essl, Klaus Peter Zulka, und Stefan Schindler. 2022. „Achieving Transformative Change in Food Consumption in Austria: A Survey on Opportunities and Obstacles“. Sustainability 14(14):8685. | Survey | Austria | 320 | - |
| Hesselberg, Julie, Susanne Pedersen, und Alice Gronhoj. 2024. „Meat Reduction Meets Family Reality: Negotiating Sustainable Diets in Households with Adolescents“. APPETITE 195:107213. doi: 10.1016/j.appet.2024.107213. | Semi-structured interviews | Denmark | 46 | 19 mothers 11 fathers 26 adolescents |
| Hielkema, Marijke Hiltje, und Thomas Bøker Lund. 2021. „Reducing meat consumption in meat-loving Denmark: Exploring willingness, behavior, barriers and drivers“. Food Quality and Preference 93:104257. doi: 10.1016/j.foodqual.2021.104257. | Survey | Denmark | 1,005 | 96.4% omnivores  2.6% vegetarians  1.0% vegans |
| Horgan, G. W., A. Scalco, T. Craig, S. Whybrow, und J. I. Macdiarmid. 2019. „Social, Temporal and Situational Influences on Meat Consumption in the UK Population“. Appetite 138:1–9. doi: 10.1016/j.appet.2019.03.007. | Secondary data analysis (National Diet and Nutrition Survey) | UK | 4,156 | - |
| Ioannidou, Maria, Valerie Lesk, Barbara Stewart-Knox, und Kathryn B. Francis. 2023. „Moral Emotions and Justifying Beliefs about Meat, Fish, Dairy and Egg Consumption: A Comparative Study of Dietary Groups“. APPETITE 186:106544. doi: 10.1016/j.appet.2023.106544. | Survey | UK | 542 | 30.8% omnivores 20.3% pescatarians 21.4% vegetarians 27.5% vegans  60.1% women  39.9% men |
| James, William H. M., Nik Lomax, Mark Birkin, und Lisa M. Collins. 2022. „Targeted policy intervention for reducing red meat consumption: conflicts and trade-offs“. BMC Nutrition 8(1):80. doi: 10.1186/s40795-022-00570-3. | Secondary data analysis (National Diet and Nutrition Survey) | UK | 5,000 | - |
| Klink, Urte, Jutta Mata, Roland Frank, und Benjamin Schüz. 2022. „Socioeconomic differences in animal food consumption: Education rather than income makes a difference“. Frontiers in Nutrition 9:993379. | Secondary data analysis | Germany | Study 1:  1,954    Study 2:  2,045 | Study 1:  51.8% women  48.2% men  Study 2:  51.7% women  48.3% men |
| Knaapila, Antti, Fabienne Michel, Kirsi Jouppila, Tuula Sontag-Strohm, und Vieno Piironen. 2022. „Millennials’ Consumption of and Attitudes toward Meat and Plant-Based Meat Alternatives by Consumer Segment in Finland“. Foods 11(3). doi: 10.3390/foods11030456. | Survey | Finland | 546  aged 20-39 years | 67.2% omnivores  12.3% flexitarians 9.5% pescatarians 4.6% vegetarians 6.4% vegans |
| Koch, Franziska, Thorsten Heuer, Carolin Krems, und Erika Claupein. 2019. „Meat consumers and non-meat consumers in Germany: A characterisation based on results of the German National Nutrition Survey II“. Journal of Nutritional science 8:e21. | Secondary data analysis (German National Nutrition Survey II) | Germany | 12,915  aged 18-80 years | 56.7% high meat consumers  41.9% low meat consumers  1.4% non-meat consumers |
| Kuosmanen, Sini, Mari Niva, Anne-Maria Pajari, Kirsi Korhonen, Toivo Muilu, und Hanna Konttinen. 2023. „Barriers Associated with Pulse and Plant-Based Meat Alternative Consumption across Sociodemographic Groups: A Capability, Opportunity, Motivation, Behaviour Model Approach“. FRONTIERS IN NUTRITION 10:1186165. doi: 10.3389/fnut.2023.1186165. | Survey | Finland | 1,000 | - |
| Laffan, Kate. 2024. „Context Counts: An Exploration of the Situational Correlates of Meat Consumption in Three Western European Countries“. BEHAVIOURAL PUBLIC POLICY. doi: 10.1017/bpp.2024.2. | Secondary data analysis  (France: INCA3,  Switzerland: National Nutrition Survey, The Netherlands: National Food Consumption Survey) | France,  Switzerland,  The Netherlands | France:  25,595  Switzerland:  19,544  The Netherlands: 26,683 | - |
| Lax, Jacob B., und Angela G. Mertig. 2020. „The Perceived Masculinity of Meat: Development and Testing of a Measure across Social Class and Gender“. FOOD CULTURE & SOCIETY 23(3):416–26. doi: 10.1080/15528014.2020.1741068. | Survey | USA | 584 | 96% omnivores  3% vegetarians  1% vegans  93% women  7% men |
| Lehto, Elviira, Niina E. Kaartinen, Katri Sääksjärvi, Satu Männistö, und Piia Jallinoja. 2022. „Vegetarians and different types of meat eaters among the Finnish adult population from 2007 to 2017“. British Journal of Nutrition 127(7):1060–72. | Secondary data analysis (FINRISK 2007, FINRISK 2012 and FinHealth 2017) | Finland | FINRISK 2007:  4,874  FINRISK 2012:  4,812  FinHealth 2017:  4,442 | FINRISK 2007:  53.9% women  FINRISK 2012:  54.6% women  FinHealth 2017:  55.4% women |
| Loginova, Daria, und Stefan Mann. 2024. „Is Eating Meat the New Smoking? Exploring the Dynamics between Meat Consumption and Education in Switzerland“. INTERNATIONAL JOURNAL OF SOCIAL ECONOMICS. doi: 10.1108/IJSE-05-2023-0335. | Secondary data analysis (Swiss Federal Statistics Office) | Switzerland | 362,871 | - |
| Ludwig-Borycz, Elizabeth, Dianne Neumark-Sztainer, Nicole Larson, Ana Baylin, Andrew D. Jones, Allison Webster, und Katherine W. Bauer. 2023. „Personal, Behavioural and Socio-Environmental Correlates of Emerging Adults’ Sustainable Food Consumption in a Cross-Sectional Analysis“. PUBLIC HEALTH NUTRITION 26(6):1306–16. doi: 10.1017/S1368980023000654. | Secondary data analysis  (EAT 2010-2018) | USA | 1,308 | - |
| Markoni, Evelyn, Thanh Mai Ha, Franziska Götze, Isabel Häberli, Minh Hai Ngo, Reto Martin Huwiler, Mathilde Delley, Anh Duc Nguyen, Thi Lam Bui, und Nhu Thinh Le. 2023. „Healthy or Environmentally Friendly? Meat Consumption Practices of Green Consumers in Vietnam and Switzerland“. Sustainability 15(15):11488. | Group discussions | Switzerland (6), Vietnam (7) | Switzerland:  42  Vietnam:  44 | - |
| Mata, Jutta, Philipp Kadel, Ronald Frank, und Benjamin Schuez. 2023. „Education- and Income-Related Differences in Processed Meat Consumption across Europe: The Role of Food-Related Attitudes“. APPETITE 182:106417. doi: 10.1016/j.appet.2022.106417. | 2 surveys | Survey 1:  Austria (5.3%), France (9.8%), Germany (20.2%), Italy (9.8%),  The Netherlands (5.0%),  Poland (9.9%), Russia (19.7%), Spain (10.0%),  UK (10.3%)  Survey 2:  France (11%), Germany (22.4%), Italy (11%),  Poland (22.3%), Russia (10.9%), Spain (11.1%),  UK (11.3%) | Survey 1:  10,226  Survey 2:  9,149 | - |
| Mertens, Alica, und Linus Oberhoff. 2023. „Meat-eating justification when gender identity is threatened – The association between meat and male masculinity“. Food Quality and Preference 104:104731. doi: 10.1016/j.foodqual.2022.104731. | Survey | Germany | 332 | 60.2% women  39.8% men |
| Mesler, Rhiannon MacDonnell, R. Bret Leary, und William J. Montford. 2022. „The Impact of Masculinity Stress on Preferences and Willingness-to-Pay for Red Meat“. APPETITE 171:105729. doi: 10.1016/j.appet.2021.105729. | Survey | UK (74.2%),  USA (12.5%),  Canada (13.2%) | 287 men | - |
| Neumann, Claudio, Samantha K. Stanley, und Diana Cárdenas. 2024. „Fleshing Out the Ways Masculinity Threat and Traditional Masculinity Ideology Relate to Meat-Eating and Environmental Attitudes in Australian Men“. Sex Roles 90(5):587–99. doi: 10.1007/s11199-024-01458-1. | Survey | Australia | 375 men | 82.4% omnivores |
| Newton, Peter, und Daniel Blaustein-Rejto. 2021. „Social and Economic Opportunities and Challenges of Plant-Based and Cultured Meat for Rural Producers in the US“. Frontiers in Sustainable Food Systems 5. doi: 10.3389/fsufs.2021.624270. | Semi-structured interviews | USA | 37 expert informants | - |
| Oleschuk, Merin, Josee Johnston, und Shyon Baumann. 2019. „Maintaining Meat: Cultural Repertoires and the Meat Paradox in a Diverse Sociocultural Context“. SOCIOLOGICAL FORUM 34(2):337–60. doi: 10.1111/socf.12500. | Semi-structured interviews | Canada | 77 | - |
| Oncini, Filippo, und Moris Triventi. 2021. „Ascent of the Herbivores, Decline of the Carnivores: The Social Stratification of Eating Profiles in Italy, 1997-2016“. POETICS 87:101533. doi: 10.1016/j.poetic.2021.101533 | Secondary data analysis (Multipurpose Survey of Daily Life by ISTAT, 1997-2016) | Italy | 481,359 | - |
| Pais, Daniel Francisco, Antonio Cardoso Marques, und Jose Alberto Fuinhas. 2023. „How to Promote Healthier and More Sustainable Food Choices: The Case of Portugal“. SUSTAINABILITY 15(4):3868. doi: 10.3390/su15043868. | Survey | Portugal | 1,040 | 81.1% omnivores  3.4% pescatarians  10.7% flexitarians  3.1% vegetarians  1.7% vegans |
| Patinho, Iliani, Erick Saldana, Miriam Mabel Selani, Ana Clara Bortoluzzi Teixeira, Beatriz Schmidt Menegali, Thais Cardoso Merlo, Juan Dario Rios-Mera, Mariana D. B. Dargelio, Heber Rodrigues, und Carmen J. Contreras-Castillo. 2021. „Original Burger (Traditional) or Burger with Mushroom Addition? A Social Representation Approach to Novel Foods“. FOOD RESEARCH INTERNATIONAL 147:110551. doi: 10.1016/j.foodres.2021.110551. | FWA task | Brazil | 209 | 58% women  42% men |
| Pechey, Rachel, James P. Reynolds, Brian Cook, Theresa M. Marteau, und Susan A. Jebb. 2022. „Acceptability of policies to reduce consumption of red and processed meat: A population-based survey experiment“. Journal of Environmental Psychology 81:101817. | Survey | UK | 2,215 | - |
| Peeters, Ouvrein, Dhoest, und De Backer. 2023. „It’s not just meat, mate! The importance of gender differences in meat consumption“. *Food, Culture and Society* 26(5):1193–1214. doi: 10.1080/15528014.2022.2125723. | Survey | UK (90.5%),  USA (7.4%) | 870 | 70.7% women  29.3% men |
| Pereira, Caroline Aparicio Dutra de Souza, Gabriela Nobre Dias, Kavita Miadaira Hamza, und Aline Ribeiro Gomes. 2023. „Changing eating habits and the impact on sustainable consumption: an analysis of the flexitarians’ journey from the perspective of Life Course Paradigm Theory“. REUNIR-REVISTA DE ADMINISTRACAO CONTABILIDADE E SUSTENTABILIDADE 13(4):113–19. | Semi-structured interviews | Brazil | 24 flexitarians | - |
| Perino, Grischa, und Claudia Schwirplies. 2022. „Meaty arguments and fishy effects: Field experimental evidence on the impact of reasons to reduce meat consumption“. Journal of Environmental Economics and Management 114:102667. | Diary study | Germany | 561 | - |
| Piracci, Giovanna, Leonardo Casini, Caterina Contini, Catalin Mihai Stancu, und Liisa Lähteenmäki. 2023. „Identifying key attributes in sustainable food choices: An analysis using the food values framework“. Journal of Cleaner Production 416:137924. doi: 10.1016/j.jclepro.2023.137924. | Survey | Italy (48.7%), Denmark (51.3%) | 1,000 | - |
| Pitt, Allison L., Jeremy D. Goldhaber-Fiebert, und Margaret L. Brandeau. 2020. „Public Health Interventions with Harms and Benefits: A Graphical Framework for Evaluating Tradeoffs“. MEDICAL DECISION MAKING 40(8):978–89. doi: 10.1177/0272989X20960458. | Case study | USA | N/A | N/A |
| Pluck, Sophie, und Angus Morrison-Saunders. 2022. „Where We Work Determines What We Eat: A Qualitative Exploration of the Multi-Dimensional Influences on Meat Consumption When Home and Office Working during the Covid 19 Lockdown in London, UK“. APPETITE 178:106147. doi: 10.1016/j.appet.2022.106147. | Semi-structured interviews | UK | 33 | 26 omnivores  3 pescatarians  3 mostly vegetarians  1 vegetarian |
| Pohlmann, Attila. 2022. „The Taste of Compassion: Influencing Meat Attitudes with Interhuman and Interspecies Moral Appeals“. APPETITE 168:105654. doi: 10.1016/j.appet.2021.105654. | 4 studies |  | Study 1: 209 Study 2: 335 Study 3: 3,431 Study 4: 87 | - |
| Randers, Louise, und John Thøgersen. 2023. „From attitude to identity? A field experiment on attitude activation, identity formation, and meat reduction“. Journal of Environmental Psychology 87:101996. doi: 10.1016/j.jenvp.2023.101996. | Field experiment, survey | Denmark | 605 pupils  aged 15-17 years | 51.2% girls  47.3% boys |
| Raptou, Elena, Amalia Tsiami, Giulia Negro, Veena Ghuriani, Pooja Baweja, Slim Smaoui, und Theodoros Varzakas. 2024. „Gen Z’s Willingness to Adopt Plant-Based Diets: Empirical Evidence from Greece, India, and the UK“. Foods 13(13). doi: 10.3390/foods13132076. | Survey | Greece (21.8%), India (58%),  UK (20.2%) | 528 born between the mid-to-late 1990s and the early 2010s | - |
| Ritzel, Christian, und Stefan Mann. 2023. „Exploring Heterogeneity in Meat Consumption and Eating out by Using a Latent Class Model“. BRITISH FOOD JOURNAL 125(1):132–44. doi: 10.1108/BFJ-11-2021-1183. | Secondary data analysis (NHANES) | USA | 41,262 | - |
| Rosenfeld, Daniel L., Hank Rothgerber, und A. Janet Tomiyama. 2020. „Mostly Vegetarian, But Flexible About It: Investigating How Meat-Reducers Express Social Identity Around Their Diets“. SOCIAL PSYCHOLOGICAL AND PERSONALITY SCIENCE 11(3):406–15. doi: 10.1177/1948550619869619. | Survey | USA | 837 | 14.2% omnivores  67.4% flexitarians  18.4% meat-excluders  56% women  44% men |
| Rosenfeld, Daniel L., und A. Janet Tomiyama. 2021. „Gender differences in meat consumption and openness to vegetarianism“. Appetite 166:105475. | Survey | USA | 1,706 | 52% women  48% men |
| Sares-Jäske, Laura, Liisa Valsta, Peppi Haario, und Tuija Martelin. 2022. „Population group differences in subjective importance of meat in diet and red and processed meat consumption“. Appetite 169:105836. doi: 10.1016/j.appet.2021.105836. | Secondary data analysis (FinHealth 2017 Study) | Finland | 4,671 aged 18-74 years | - |
| Serrano-Balcázar, Mireya Cristina. 2020. „Per capita income as a determinant of meat consumption in three cities in China“. Polo del Conocimiento: Revista científico-profesional 5(5):275–95. | Survey | China | 356 | Shanghai: 111 Yichang: 137 Xi'an: 108 |
| Stanley, Samantha K. 2022. „Ideological Bases of Attitudes towards Meat Abstention: Vegetarianism as a Threat to the Cultural and Economic Status Quo“. Group Processes & Intergroup Relations 25(6):1534–54. doi: 10.1177/13684302211020356. | 2 surveys | Study 1:  Australia  Study 2:  USA | Study 1:  197  Study 2:  453 | Study 1: 64.5% omnivores  20.3% flexitarians  10.7% vegetarians  4.6% vegans  Study 2:  78.9% omnivores  13.6% flexitarians  5.7% vegetarians  1.8% vegans |
| Szejda, Keri, Moritz Stumpe, Ludwig Raal, und Claire E. Tapscott. 2021. „South African Consumer Adoption of Plant-Based and Cultivated Meat: A Segmentation Study“. FRONTIERS IN SUSTAINABLE FOOD SYSTEMS 5:744199. doi: 10.3389/fsufs.2021.744199. | Survey | South Africa | 1,087  aged 18-61 years |  |
| Tschanz, Linda, Ivo Kaelin, Anna Wróbel, Sabine Rohrmann, und Janice Sych. 2022. „Characterisation of Meat Consumption across Socio-Demographic, Lifestyle and Anthropometric Groups in Switzerland: Results from the National Nutrition Survey menuCH“. Public Health Nutrition 25(11):3096–3106. doi: 10.1017/S136898002200101X. | Secondary data analysis (menuCH) | Switzerland | 2,057  aged 18-75 years | 54,6% women  45,4% men |
| van den Berg, Saskia W., Annelien C. van den Brink, Annemarie Wagemakers, und Lea den Broeder. 2022. FOOD QUALITY AND PREFERENCE 100:104623. | Survey | The Netherlands | 1,670 | 27.2% high meat 36% middle meat  29.3% low meat  7,4% vegetarians |
| Wendler, Morten. 2023. „The Social Challenges of Not Eating Meat: How Social Interactions Shape the Role of Meat in Everyday Food Practices“. Consumption and Society 2(1):24–41. doi: 10.1332/HJWX1794. | Focus groups and semi-structured interviews | Denmark | 27 aged 20-29 years | - |
| Willits-Smith, Amelia, Harmonii Odinga, Keelia O’Malley, und Donald Rose. 2023. „Demographic and Socioeconomic Correlates of Disproportionate Beef Consumption among US Adults in an Age of Global Warming“. NUTRIENTS 15(17):3795. doi: 10.3390/nu15173795. | Secondary data analysis (NHANES, 2015-2018) | USA | 10,248 | 51.5% women  48.5% men |
| Wolfson, Julia A., Amelia M. Willits-Smith, Cindy W. Leung, Martin C. Heller, und Donald Rose. 2022. „Cooking at Home, Fast Food, Meat Consumption, and Dietary Carbon Footprint among US Adults“. INTERNATIONAL JOURNAL OF ENVIRONMENTAL RESEARCH AND PUBLIC HEALTH 19(2):853. doi: 10.3390/ijerph19020853. | Secondary data analysis (NHANES, 2007-2010) | USA | 11,469 | - |
| Wolfswinkel, Sofia, Sanne Raghoebar, Hans Dagevos, Emely de Vet, und Maartje P. Poelman. 2024. „How Perceptions of Meat Consumption Norms Differ across Contexts and Meat Consumer Groups“. APPETITE 195:107227. doi: 10.1016/j.appet.2024.107227. | Survey | UK | 1,205 | 33% meat lovers 8.6% exceeders 13.1% flexitarians 45.3% moderates |

Notes: N/A = not applicable because data other than those collected from human subjects were used.

**
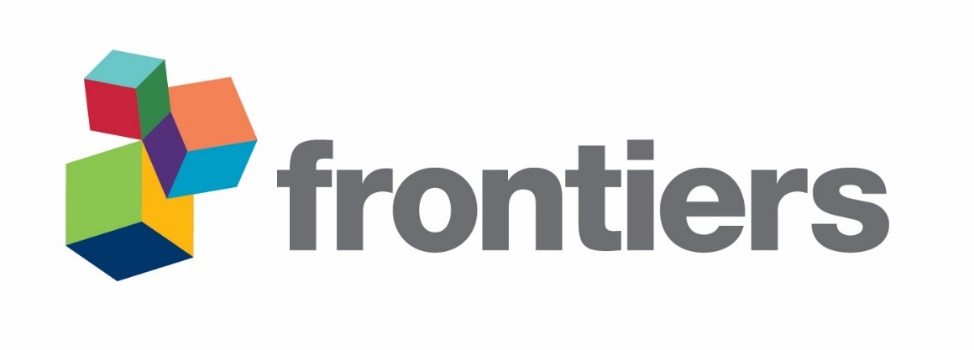
**
